# Staff perspectives of a model of access and triage for reducing waiting time in ambulatory services: a qualitative study

**DOI:** 10.1186/s12913-019-4123-0

**Published:** 2019-05-03

**Authors:** Katherine E. Harding, David A. Snowdon, Annie K. Lewis, Sandra G. Leggat, Bridie Kent, Jennifer J. Watts, Nicholas F. Taylor

**Affiliations:** 1 Allied Health Clinical Research Office, Eastern Health, Level 2/5 Arnold Street, Box Hill, VIC 3128 Australia; 2 La Trobe University, Kingsbury Drive, Bundoora, VIC 3086 Australia; 3 Plymouth University, Drake Circus, Plymouth, Devon PL4 8AA UK; 4 Deakin University, 221 Burwood Highway, Burwood, VIC 3125 Australia

**Keywords:** Waiting lists, Access, Appointments and schedules, Outpatients, Community health, Clinician experience, Qualitative

## Abstract

**Background:**

Specific Timely Appointments for Triage (STAT) is an intervention designed to reduce waiting time in community outpatient health services, shown to be effective in a large stepped wedge cluster randomised controlled trial. STAT combines initial strategies to reduce existing wait lists with creation of a specific number of protected appointments for new patients based on demand. It offers an alternative to the more traditional methods of demand management for these services using waiting lists with triage systems. This study aimed to explore perceptions of clinicians and administrative staff involved in implementing the model.

**Method:**

Semi-structured interviews with 20 staff members who experienced the change to STAT were conducted by an independent interviewer. All eight sites involved in the original trial and all professional disciplines were represented in the sample. Data were coded and analysed thematically.

**Results:**

Participants agreed that shorter waiting time for patients was the main advantage of the STAT model, and that ongoing management of caseloads was challenging. However, there was variation in the overall weight placed on these factors, and therefore the participants’ preference for the new or previous model of care. Perceptions of whether the advantages outweighed the disadvantages were influenced by five sub-themes: staff perception of how much waiting matters to the patient, prior exposure to the management of waiting list, caseload complexity, approach and attitude to the implementation of STAT and organisational factors.

**Conclusions:**

The STAT model has clear benefits but also presents challenges for staff members. The findings of this study suggest that careful preparation and management of change and active planning for known fluctuations in supply and demand are likely to help to mitigate sources of stress and improve the likelihood of successful implementation of the STAT model for improving waiting times for patients referred to community outpatient services.

## Introduction

Patients referred to publicly-funded community outpatient services often face long waiting times for treatment [1–4]. These multi-disciplinary outpatient services provide assistance and support for people who are recovering from illness or injury, managing chronic diseases or living with disabilities in the community*.* Individual community outpatient services may focus on providing care for a specific health problem (for example a continence or dementia clinic) or at a specific stage of recovery (such as a community rehabilitation program). Care is usually provided over multiple appointments with medical, nursing or allied health professionals and the services can often be an important adjunct to hospital-based services, both by preventing hospital presentations and supporting discharge back to the community after a hospital stay.

Given that the conditions of the patients treated by these services are often chronic or sub-acute in nature, wait lists organised by triage systems are commonly used to manage demand [5, 6]. New patients are assessed for eligibility, categorised according to priority and placed on a wait list until capacity is available to accept them into the service. The disadvantages of this “waitlist and triage” include poor reliability of triage tools, normalising of long wait periods, diversion of resources from clinical care to management of the waiting list and the very real possibility that low priority clients will never be seen [6]. Furthermore, as reported by others in primary care [7], it is not uncommon to observe situations in services using this model of access where waiting times have been constant for very long periods of time, suggesting that supply and demand are actually in balance but the service constantly operates with a backlog of waiting patients.

Specific Timely Appointments for Triage (STAT) is a model of access and triage that was designed as an alternative to the traditional “waitlist and triage” approach with the aim of reducing waiting time for community outpatient health services [8]. STAT begins with a single, targeted intervention to reduce the existing waiting list. Service demand is then carefully calculated and the specific number of new assessment appointments required to keep up with demand is protected in clinician schedules. New patients are booked immediately into an assessment appointment on referral, providing timely access to the service. The treating clinician combines initial assessment and triage, making priority decisions about ongoing service needs based on their clinical expertise and in the context of their existing caseload. Web-based resources providing a detailed description of the intervention are freely available for download [9].

STAT reduced waiting times in trials conducted in community rehabilitation and outpatient physiotherapy [8, 10]. More recently, a stepped wedge cluster randomised controlled trial was conducted involving 3116 patients referred to eight sites providing community outpatient services within a large metropolitan health service [11]. The primary outcome of time from referral to first appointment reduced from a median of 42 days to a median of 24 days, a 34% reduction attributable to the intervention after controlling for clustering by service. Variability in waiting time was also substantially reduced, suggesting that the greatest benefit of the model was likely to be for patients who were previously judged to be low priority and waited for long periods to access care. This trial was registered with the Australian and New Zealand Clinical Trials Registry (ACTRN12615001016527) and has been reported in detail elsewhere [12].

While these findings are encouraging, whether the model is sustainable over the longer term depends partly on whether it is acceptable to staff within the services. Sekhon defines acceptability as the “extent to which people delivering or receiving a healthcare intervention consider it to be appropriate”, and emphasises the importance of robust research into acceptability when evaluating healthcare interventions [13]. Furthermore, human factors, including engagement and support from program leaders and staff have been widely recognised to be key factors in sustainability of health service interventions [14–16]. A previous qualitative evaluation with staff involved in implementing STAT at a single community rehabilitation program suggested that participants found the start-up period challenging, but were generally supportive of the model 6 months after it was embedded into practice [17].

The current stepped wedge cluster randomised controlled trial provided an opportunity to investigate the experience of staff in a broader range of services. This study therefore aimed to explore staff perceptions of the implementation process, the impact of the STAT model on staff workload and patient care, and overall acceptability of the model in eight different sites providing a variety of health services within community outpatient settings.

## Method

### Design

A qualitative study design was used following an interpretive description approach [18] and reported in accordance with the COREQ checklist [19]. In-depth semi structured interviews were conducted to explore the experiences of staff who were involved in the implementation of the STAT model. The aims of the interview were to establish staff perceptions on (1) what aspects of STAT worked well, (2) what aspects had been challenging or failed, (3) how the implementation process could be improved, (4) how much implementation of STAT changed daily practice, (5) the effect of STAT on patient outcomes and (6) the overall perception of the model relative to usual care. All interviews were conducted between July and October 2017, following the end of the STAT trial [12]. This qualitative study received ethics approval from the Eastern Health Human Research Ethics Committee as an amendment to the approval previously obtained for the stepped wedge trial. All participants completed written informed consent.

### Participants

Participants were recruited from a large metropolitan health service in Victoria, Australia, consisting of several inpatient facilities providing acute and subacute care as well as two smaller satellite sites providing community-based care. Clinicians and administration staff providing outpatient and/or community care directly involved in the implementation of the STAT model of access and triage (*n* = 47) were eligible to participate in the study. Senior managers who did not have direct contact with patients were not included. Eligible staff were invited via email to express their interest in participating, and purposive sampling was then used to select 20 participants with the aim of ensuring that all eight trial sites, professional groups and levels of seniority were represented among the participant group. This ‘maximum variation’ approach to sampling is widely used to obtain an ‘information rich’ sample, and is particularly appropriate when the variation within the population of interest is well understood [20]. The sample size of 20 represented of 43% of the eligible population, and was expected to be sufficient to reach data saturation.

### Data collection

Interviews were conducted face to face by a female interviewer with an allied health background (Bachelor of Occupational Therapy) who had neither previous history with the services involved in the study nor prior involvement in the planning or implementation of the STAT trial. The choice of interviewer aimed to achieve credibility while maintaining sufficient independence to minimise the possibility of social desirability bias. All interviews took place in a private room at the participants’ workplace. They ranged from 20 min to 1 h in length and were audio recorded and transcribed verbatim. A semi-structured interview guide (Table 1) was used to ensure that all relevant topics of interest regarding the experiences of the participants’ implementation of the STAT model were addressed. The transcriptions were then provided to the participants to ensure that the documents were an accurate representation of the participant’s perceptions [21]. Participants were encouraged to make amendments to the transcripts if they believed that the transcript did not portray what they intended to say.

**Table 1:** Interview Schedule

Topic Area	Sample questions
Introduction	Can you briefly describe the work that you do? Can you describe the changes that were made to your service?
Pre intervention	Can you describe if there were things about the service that needed to be improved? What was the “case for change”? Did you perceive waiting times to be a problem in this service?
Implementation period	How would you describe your experience during the implementation period? What worked well? What was difficult?
Effect of the model on staff and work practices	Now that the new model is in place, how do you find it to work with compared to the traditional model? How does the new model affect workload? What methods do you use to manage your caseload? Have you changed the way that you schedule patients for ongoing treatment? Has the model led you to identify inefficiencies that you didn’t notice before? Are there other processes that have become more efficient?
Effect on patient care	What effect, if any, do you feel the model had on patient care? Do you think that there have been benefits to your patients? Do you think that any patients have been disadvantaged in any way?
Overall opinion/ future direction	Can you describe any other benefits of the new system? Can you describe any other disadvantages? If you were to be in the position of being a manager overseeing the introduction of this model in the future, is there anything that you would do differently? As a staff member of the service, which model would you prefer to use in your workplace? If you had a family member seeking treatment at this service, which model would you prefer the service to be using? Why?

### Data analysis

Transcripts were analysed thematically using an inductive approach. Each transcript was independently coded with the assistance of a software package for qualitative data analysis by two researchers (Nvivo version 10; QSR International). Two additional members of the research team read all transcripts and provided an overview impression. The four researchers then collaborated, discussing similarity between the codes and overall impressions until consensus on the major themes and sub-themes was achieved.

Finally, a matrix analysis was performed to examine differences between the views and experiences of participants who expressed an overall preference for the STAT model and those who preferred working in the way they had done previously [22].

## Results

Twenty staff members, including 16 clinical and four administrative staff took part in the study (Table 2). All eight trial services were represented by at least one team member involved in the implementation of the STAT model. All participants were female, reflecting the overall profile of the workforce at the eight sites of which more than 90% are female. Participating clinicians were all at least Grade 2 (mid-career level, with a minimum of 5 years clinical experience) and 3 had team leadership responsibilities in addition to clinical roles. This also reflects the workforce in these services which rarely offer roles for new graduate or junior staff. No new codes were introduced during the analysis of the final transcriptions suggesting that saturation had been reached.

**Table 2:** Summary of participants (*n* = 20) and their services

	number
Employing service type	
Community health service	8
Adult	3
Paediatric	5
Multi-disciplinary specialist clinic	8
Physiotherapy Outpatient clinic	4
Profession of participants	
Administration	4
Allied Health Assistant	1
Nurse	2
Occupational Therapist	2
Physiotherapist	8
Speech Pathologist	3
Geographical location of service	
Metropolitan	12
Rural/semi-rural	8
Seniority of participants	
Team leader	3
Clinician (mid-career level)	12
Administrative/assistant role	5

**Fig. 1:**
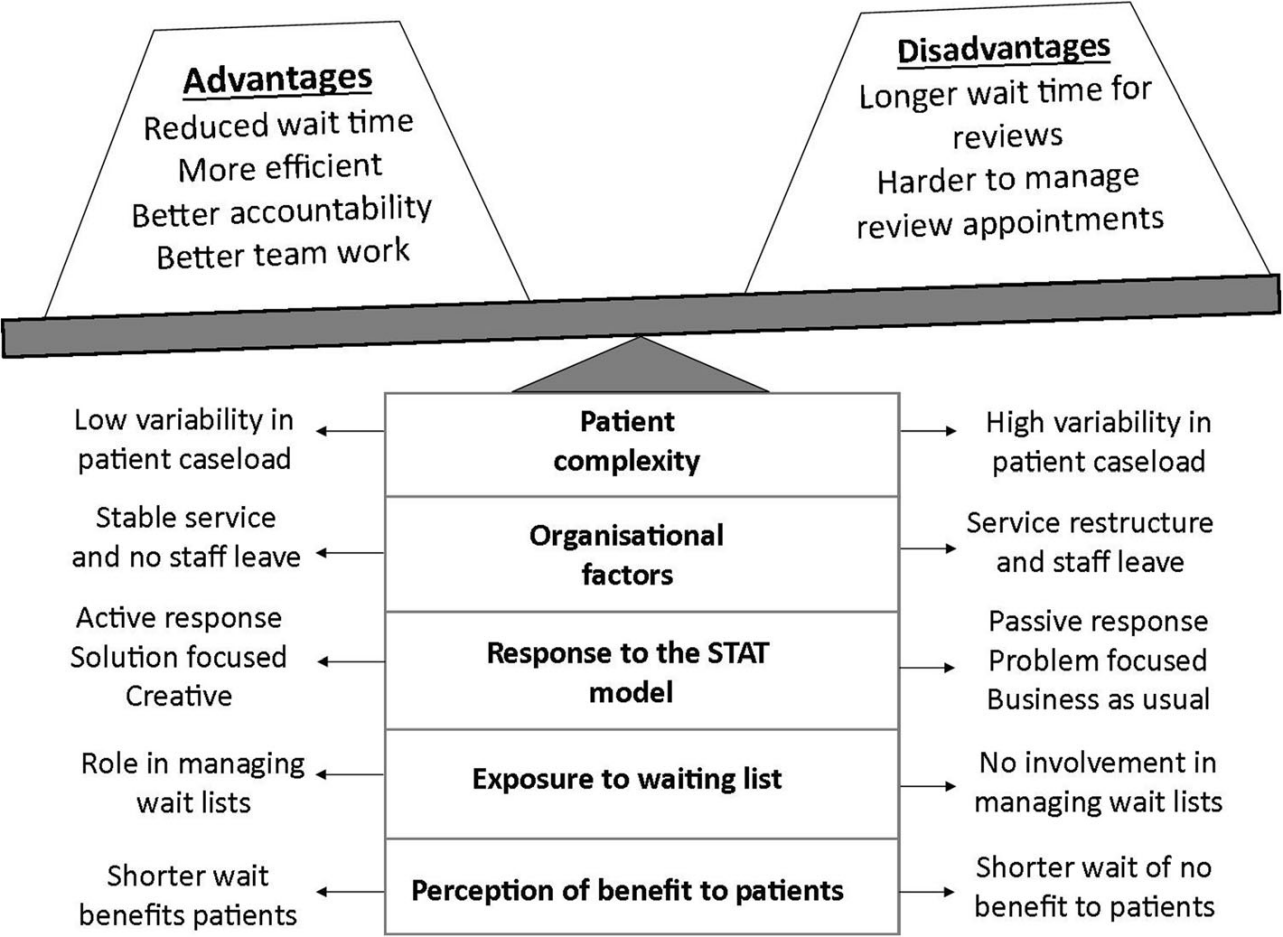
Conceptual model of factors influencing participants’ views about the relative weight given to the advantages and disadvantages of the STAT model

Participants broadly agreed about the advantages and disadvantages of the STAT model, but there was variation in their response to the overall question, “Which model would you prefer to use in your workplace?” Thirteen participants expressed a clear preference for STAT. The remaining seven all recognised some benefits in the model, but expressed reservations about its ongoing use or sustainability in their service. These preferences did not appear to be distributed according to site. The participants with reservations were from five different sites, four of which also had participants who were in favour of the STAT model. Five sub-themes emerged that appeared to influence whether participants favoured the STAT model over the pre-existing model of access and triage for new patients: perception of benefit to patients; exposure to waiting list; response to the STAT model; organisational factors; and patient complexity (Fig. 1).

### Advantages and disadvantages of the STAT model

Participants’ identified several advantages to the implementation of the STAT model. First, participants reported that STAT significantly reduced the wait time for patients who were referred to their service:

*It feels faster paced. We’re being more responsive. We’re seeing clients quicker. We’re meeting their needs faster and being involved with them sooner rather than later. (P17)*

To achieve this reduction in wait times, participants commented that the STAT model ensured accountability of all team members in ensuring that the wait times were short and patients on waiting lists were prioritised:

*That was quite powerful in that, they got their times, they got them booked and we were committed to having those times for them. I think it kept us quite accountable too. (P10)*

Furthermore, in the process of reducing wait times, team members of the participating services reported having worked more collaboratively and effectively as a team.

*I think we work better as a team. You know it’s really brought us together more and I think we work more effectively. (P18)*

In addition to reducing wait times, the STAT model introduced new efficiencies in the processing of new referrals:

*It is definitely a lot more efficient processing referrals…All those processes have been more streamlined. (P17)*

The time saved not triaging new patients was also appreciated by participants:

*We certainly saved time with not having to prioritise [new referrals before making appointments]. (P9)*

In contrast to the advantages of STAT, participants reported disadvantages relating to the challenges of prioritising and reviewing patients following their initial appointment. Longer times to patients’ first review appointment were a common concern for participants:

*The only thing is that they’re seen quicker for their initial but they might wait longer for their review, because we’re getting more people seen there’s more people to schedule in. (P17)*

Participants reported difficulty scheduling review appointments in their diaries, which were populated with new assessments:

*When someone has tried to look at a review appointment slot, sometimes they can’t be booked in for 6-8 weeks because the diary is already populated. (P13)*

Some also reported that once they became aware of all the patients requiring their services, they had difficulty prioritising patients for ongoing management. This led to some clinicians having a higher number of patients on their caseloads post-implementation of STAT:

*The whole idea is that we see the client; we prioritise them from their initial assessment and then review them or treat them down the track. But that’s been very challenging for me…once they’re on your caseload you have this emotional burden of them. Instead of being a name on a list that you call when you get a chance. (P10)*

### Factors that affected participants’ overall view of the STAT model compared to previous practice

Although most participants broadly agreed on the advantages and disadvantages of the STAT model, their overall view on whether they favoured the STAT model over previous models of triaging patients appeared to be influenced by five sub-themes.

#### Perception of benefit for patients

Participants who favoured the STAT model were more likely to report that patients benefited from a short wait time:

*Yeah I do think that the benefits of being seen earlier speak for themselves. You can improve their health outcomes sooner rather than later and that’s better for everybody. (P7)*

In contrast, participants who favoured previous models of triage were more likely to report no benefit to patients who wait shorter times for their initial appointment:

*Often the problems they’ve had for many, many years, so the wait didn’t seem to be such a big problem. (P15)*

#### Exposure to waiting list

Participants who had direct responsibility for managing the wait list prior to the introduction of STAT were more likely to recognise the benefits of new efficiencies:

*I’ve got other work that I’m doing now which is more constructive for the team. I’m not just sitting there managing a wait list. (P20)*

Those with less responsibility for the wait list prior to the implementation of STAT, were less likely to view the wait time as a problem that needed to be addressed:

*They were waiting between 8-14 weeks. That’s sort of been a stock standard waiting time over the last three or four or five years. (P13)*

#### Response to STAT model

Some participants demonstrated a more active response to the introduction of STAT than others, developing creative solutions to prioritise and manage their caseloads. Those who responded to the change in a more active way were more likely to have a positive view of the model:

*You might just have to be a bit more creative with your session, showing them how they can progress a bit further independently…But the pro is that we are seeing more people sooner, which I think is more of a priority. (P3)*

Conversely, those less in favour of the model often described a more passive approach to the implementation of the STAT model, and appeared less inclined to seek innovative solutions to the challenges STAT presented:

*I have to spend half my time triaging. That hasn’t changed with the STAT project. (P5)*

#### Organisational factors

Organisational factors outside of the control of individual staff members were a challenge at some of the services. Factors such as unplanned leave or other service disruptions added complexity to the introduction of the model at some sites, in contrast to others that were operating in a more stable environment.

*It’s the availability of cover for unplanned leave. There is very little cover and the late cancellations have a big impact. (P12)*

Participants working at services with more stability were more likely to report benefits, and in some cases, reported that STAT assisted in meeting demands during times of staff shortages:

*It’s definitely decreased the waiting times, particularly during busy periods. When someone’s sick we can get the client in a lot sooner. (P4)*

#### Patient complexity

The complexity of the client caseload at the various services also appeared to influence the response of the staff, although participants did not always agree about whether the STAT model was better or worse for patients with complex needs. Some reported concern that their highly complex patients were at risk of deteriorating during an increased wait for review time, whereas others believed that longer wait times for the initial appointment were a greater risk:

*I still think that you’re putting patients more at risk that have had to wait three weeks and haven’t had contact. I feel comfortable to know that we have seen someone at least and been able to get them started on their rehab. (P3)*

Some participants reported that the patients they manage are too complex to change their model of care, and expressed doubt about the applicability of the model to their setting:

*I find rehabilitating a knee replacement or a hip replacement is a lot more straightforward than some of these patients. They’re complex, there are lots of issues feeding in. (P14)*

## Discussion

Clinicians and administrative staff who were directly involved in the implementation of the STAT model agreed that it improved access for patients, but led to some challenges, particularly in scheduling review appointments. Perception of benefit to patients, prior exposure to the waiting list, response to the model, organisational factors and patient complexity appeared to influence whether clinicians would choose this model over the way they had previously managed service demand.

The perceived advantage of reduced waiting times concurs with the main findings from the stepped wedge cluster randomised controlled trial, which found an average reduction in waiting of 34% (24 days) [12]. In contrast, the perception of an increased wait time to first review appointment is not supported by findings in the trial [12]. While it is possible that there were individual cases where clinicians had difficulty scheduling patients to their first review appointment, there was no difference recorded in the time between the first and second appointment after the STAT model had been implemented across the eight sites. The perception of an increased wait time to the first review appointment may be due to the stresses of adjusting to the new model and a feeling of lack of control by clinicians over their schedules. The option for clinicians to close their diaries to new patients and use the majority of appointments slots to treat existing patients was no longer available to them during times of high demand [11]. Instead, clinicians needed to accept that their review appointments were in limited supply and they needed to make priority decisions when allocating these resources. Those who embraced the change and saw the new model as an opportunity to streamline their service were less likely to report concerns about review appointments.

The findings of the current study suggest that there were more clinicians who maintained some reservations about the STAT model compared to a previous qualitative study of staff perceptions of STAT conducted within a single community rehabilitation service [17]. Clinicians in the earlier study spoke of early scepticism and challenges during the changeover period to the STAT model, but all were either neutral in their opinion or reported a preference for STAT at the time of interview. These clinicians also spoke about the importance of effectively managing the change process. Both qualitative studies took place alongside a clinical trial, and one reason for the differences in findings between the two cohorts may lie in the trial design. The first was a controlled before and after trial involving a single site and some flexibility with timing of the intervention. The second was a stepped wedge cluster randomised controlled trial that required implementation timing to comply with a strict schedule. This is a recognised challenge of this trial design [23] and allowed little opportunity for flexibility if staff needed longer to understand the case for change and adjust their practice. In addition, the decision to participate in the stepped wedge trial was made by service managers, resulting in an external rather than internal driver for the intervention that may have contributed to difficulty in achieving full implementation at some sites [24]. These factors would be less likely to affect services implementing STAT outside of the context of a research trial. However, these observations do reinforce the importance of careful attention to established principles of good change management in implementing service level interventions.

Considering results from this study in the context of implementation science literature provides insights into measures that could be taken to mitigate clinician concerns and improve the prospect of sustainability over the long term. First, a perception by stakeholders that an intervention has a clear “relative advantage” over existing practices increases the likelihood of successful implementation [24]. In the current study, consistent with the construct of observability [25], staff who had more direct knowledge of the waitlist appeared more likely to value the benefits of the STAT model, suggesting that ready access to these data for all staff may be an important component of implementation. Also providing staff with clear evidence statements about the effectiveness of STAT in reducing waiting time may enhance perception of the relative advantage of this over other methods of managing access and triage processes [24]. Second, sufficient attention must be given to adapting the intervention to local settings [24]. Some participants reported the process of prioritising review appointments to be challenging, and this may be a reflection of insufficient attention given to adaptation. Providing opportunities for teams to generate ideas to address the specific challenges of their local setting and the support to test them may be another strategy to address the concerns raised by the participants in this study. Since the conclusion of the trial, further resources have become available that explain the model and provide a step by step guide to implementation, which may also assist this process [9]. Finally, anticipating and planning for predictable fluctuations in supply and demand can help to ensure that the service remains stable, therefore enabling change by removing barriers [26]. For example, planning ahead for predictable disruptions in supply (such as maternity leave) or reserving resources to cope with seasonal fluctuations in demand.

The perception of some health care providers that long wait times for services have little impact on patient health outcomes is likely to contribute to increased wait times [27]. Results from this study add further weight to this argument, as this perception also appears to have been a barrier to acceptance of the STAT model by some participants. However, a systematic review involving 14 studies found that delays in access to community outpatient services were associated with poorer health outcomes and workplace participation [28]. Excessive waiting times for chronic pain have also been found to be associated with adverse health effects during the waiting period [29].

This study is limited to the perceptions of 20 participants who experienced the implementation of the STAT model at one of eight sites. Although data saturation appeared to be reached with no new themes arising in the final interviews, there was considerable variation in the relative importance that the participants placed on different factors in forming an overall view about the model. It is possible that inclusion of a wider range of people may have led to more of a consensus. However, the sample was able to provide a rich data set that fulfilled the purpose of the study which was to explore the issues for staff in implementing the model.

The interviews in this study were all conducted by a researcher from outside of the organisation who was not involved in the stepped wedge cluster randomised controlled trial, and had never had any previous experience in the STAT model. Overall this is a strength of the study, as there was likely to be little influence of confirmation bias or social desirability bias. Lack of direct prior experience with STAT from the interviewer may, however, have limited opportunities to challenge participants or explore inconsistencies in relation to stressors that participants associated with the trial period but were unrelated to the STAT implementation. Therefore some questions remain about the degree to which the responses in this study were driven by the model itself, and how much they were influenced by the process of implementation. Further research is needed to better understand these issues.

## Conclusion

The STAT model reduced waiting time in a stepped wedge cluster randomised controlled trial involving eight community outpatient health services. This qualitative evaluation of staff perceptions suggests that those working with the model agree that timely access for patients is a benefit of STAT, but managing review appointments can be challenging. Whether the balance between these competing factors leads to overall support for the service is influenced by views about waiting for care, prior exposure to the waiting list, clinician approaches to change, caseload complexity and organisational factors. The findings suggest that taking time to build a case for change, supporting clinicians and administration staff to adapt to the process of actively prioritising caseload demands, and taking deliberate steps to plan for known fluctuations in supply and demand are likely to improve the likelihood of success for services planning to implement the STAT model to reduce waiting times for patients referred to community outpatient services.

## Abbreviations

STAT: Specific Timely Appointments for Triage
